# Urinary copper at 1 month predicts early neurological worsening in treatment-naïve Wilson’s disease on ultra-low dose penicillamine

**DOI:** 10.3389/fneur.2026.1946980

**Published:** 2026-10-26

**Authors:** Yue Luo, Mansi Cai, Jie Yang, Xiangxue Zhou, Chao Wu, Huajing You, Xunhua Li, Dingbang Chen

**Affiliations:** 1 Department of Neurology, The First Affiliated Hospital, Sun Yat-sen University, Guangdong Provincial Key Laboratory of Diagnosis and Treatment of Major Neurological Diseases, National Key Clinical Department and Key Discipline of Neurology, Guangzhou, China

**Keywords:** penicillamine, plasma NFL, serum exchangeable copper, urinary copper, Wilson’s disease

## Abstract

**Background:**

Penicillamine can trigger early neurological worsening in patients with Wilson’s disease (WD). We evaluated a very conservative regimen, starting at 125 mg/day and increasing the does stepwise at months 1, 3, and 6, combined with zinc, focusing on clinical outcomes, urinary copper, exchangeable copper (CuEXC), and plasma neurofilament light (NfL).

**Methods:**

We retrospectively analyzed 25 treatment-naïve patients with neurological WD who received penicillamine at 125 mg/day, with 125-mg/day increments at months 1, 3, and 6, plus zinc gluconate. Neurological status (UWDRS), 24-h urinary copper, CuEXC, and NfL were assessed at baseline and at 1, 3, 6, and 12 months. Neurological worsening was defined as an increase of more than 2 points in the UWDRS-N.

**Results:**

Nine patients (36%) experienced neurological worsened, all within the first 3 months. Patients who remained stable (*n* = 16) showed progressive improvement in UWDRS scores and a sustained decline in CuEXC. Patients who experienced worsening had a blunted increase in urinary copper at 1 month and no significant reduction in CuEXC. ROC curve analysis showed that a 1-month urinary copper level above 473.85 μg/day was associated with a lower risk of worsening. Plasma NfL showed a mild, transient elevation in both groups at 1 month, followed by a decline only in the stable groups; however, between-group differences were not statistically significant.

**Conclusion:**

Neurological worsening remains frequent (36%) despite the ultra-low starting doses. A simple 1-month urinary copper threshold of 473.85 μg/day may help identify patients with more favorable outcomes. CuEXC and NfL changes parallel clinical courses but require larger validation.

## Introduction

Penicillamine is a first-line chelator for Wilson’s disease (WD), but early neurological deterioration complicates 10–50% of treatment initiations, particularly when high doses are used (1–3). To mitigate this risk, guidelines recommend gradual dose escalation from 250 to 500 mg/day (4, 5); however, no consensus exists regarding the optimal starting dose or titration rate. Even with low-dose protocols, neurological worsening is not eliminated, and objective early predictors remain lacking.

We adopted an even more conservative regimen, starting at 125 mg/day with gradual dose increments, and combined it with zinc to minimize early deterioration. In this brief report, we retrospectively analyzed clinical outcomes and longitudinal changes in three biomarkers—24-h urinary copper, serum exchangeable copper (CuEXC), and plasma neurofilament light (NfL)—in 25 treatment-naïve patients with neurological WD. The primary objective was to characterize early neurological outcomes after initiating an ultra-low-dose penicillamine regimen and to explore early biochemical biomarkers associated with subsequent neurological deterioration.

## Methods

### Study design and participants

This was a single-centre, retrospective observational study. We reviewed the medical records of all treatment-naïve patients with neurological WD who attended the First Affiliated Hospital of Sun Yat-sen University between July 2020 and February 2023 and received the fixed low-dose protocol. The diagnosis was established according to the Leipzig criteria (1). Patients were excluded if they had received prior chelation therapy, lacked baseline UWDRS data, or had no 1-month urinary copper measurement. Twenty-five eligible patients were identified.

### Treatment

The initial penicillamine escalation schedule followed a predefined, fixed protocol. Oral penicillamine was initiated at 125 mg/day, with increases of 125 mg/day at months 1, 3, and 6 reaching a target dose of 500 mg/day by month 6. Discontinuation or switching of treatment was at the discretion of the attending physician in the event of neurological deterioration. All patients received concomitant zinc gluconate (50 mg elemental zinc three times daily) because it is well-established to reduce intestinal copper absorption. Because zinc was administered to all patients in the cohort, its independent effect on neurological outcomes could not be evaluated separately.

### Data collection

Clinical and laboratory data were extracted from electronic medical records at baseline and at scheduled follow-ups (1, 3, 6, and 12 months): UWDRS (neurological subscale, UWDRS-N; psychiatric subscale, UWDRS-P; hepatic subscale, UWDRS-H; total), 24-h urinary copper, serum CuEXC (measured by EDTA-ultrafiltration followed by ICP-MS), and plasma NfL (measured using the Simoa HD-X platform). Not all patients had complete CuEXC or NfL measurements at every time point due to the retrospective nature; available data were analyzed. Neurological worsening was defined as an increase in UWDRS-N >2 points from baseline, as previously described (4). This study was approved by the Institutional Ethics Committee ([2020]056); written informed consent was waived for this retrospective chart review.

### Statistical analysis

Continuous variables are presented as median (IQR) and compared by Mann–Whitney U test (between groups) or the Wilcoxon signed-rank (within group). Categorical variables were analyzed using Fisher’s exact test. No adjustment for multiple comparisons was applied given the exploratory design. A *p* value of less than 0.05 was considered statistically significant. Analyses were performed using SPSS version 25.0.

## Results

### Patient characteristics and clinical outcomes

Of the 25 patients, 9 (36%) met the criterion for neurological worsening, all within the first 3 months (5 at month 1 and 4 at month 3). Baseline characteristics are presented in Table 1. Patients who experienced worsening had significantly higher baseline UWDRS-P scores than stable patients (10.0 vs. 3.0, *p* = 0.033) and a higher prevalence of limb hypertonia (55.6% vs. 6.3%, *p* = 0.012), indicating greater baseline impairment in these measures.

**Table 1:** Comparison of baseline characteristics between the stable group and the worsening group.

Index	Stable group (*n* = 16)	Worsening group (*n* = 9)	*p*-value
Gender (male/female)	12/4 (75.0%/25.0%)	7/2 (77.8%/22.2%)	0.843
Onset age (year)	23 0.0 (10.0–46.0)	19.2 (17.0–24.0)	0.832
Duration of disease (months)	15.0 (1–36)	8.8 (1–24)	0.881
UWDRS-N score	13.00 (6.00–16.00)	20.00 (14.00–27.50)	0.060
UWDRS-H score	1.00 (0.00–3.00)	2.00 (0.00–3.50)	0.424
UWDRS-P score	3.00 (2.00–5.00)	10.00 (3.50–16.00)	0.033
Total UWDRS score	20.40 (6–58)	32.11 (11–48)	0.064
24-h urinary copper (μg/day)	311.20 (60.01–493.70)	431.90 (144.84–630.14)	0.846
Serum CuEXC (μg/L)	82.61 (53.63–117.65)	61.59 (35.93–123.18) μg/L	0.940
Plasma NfL (pg/mL)	14.61 (5.03–65.22)	15.04 (7.21–35.37)	0.764

In the stable group (*n* = 16), total UWDRS declined from 20.4 (6–58) at baseline to 8.6 (6–26) at 12 months (*p* = 0.039). In the worsened group, total UWDRS rose from 32.0 (11–48) to 37.8 (20–52) at 3 months (*p* = 0.119), after which most patients were switched to alternative chelators.

### Urinary copper excretion

At 1 month, stable patients excreted significantly more copper than worsened patients: 901.5 (481–1,697) vs. 596.8 (432–788) μg/d, *p* = 0.032 (Figures 1a,b). ROC curve analysis showed that 24-h urinary copper had some diagnostic performance for disease worsening, with an AUC of 0.830. The optimal cutoff value was 473.85 μg**/**day, with a sensitivity of 77.8% and a specificity of 86.7%; the positive predictive value was 0.700, and the negative predictive value was 0.857 (Figure 2).

**Figure 1:**
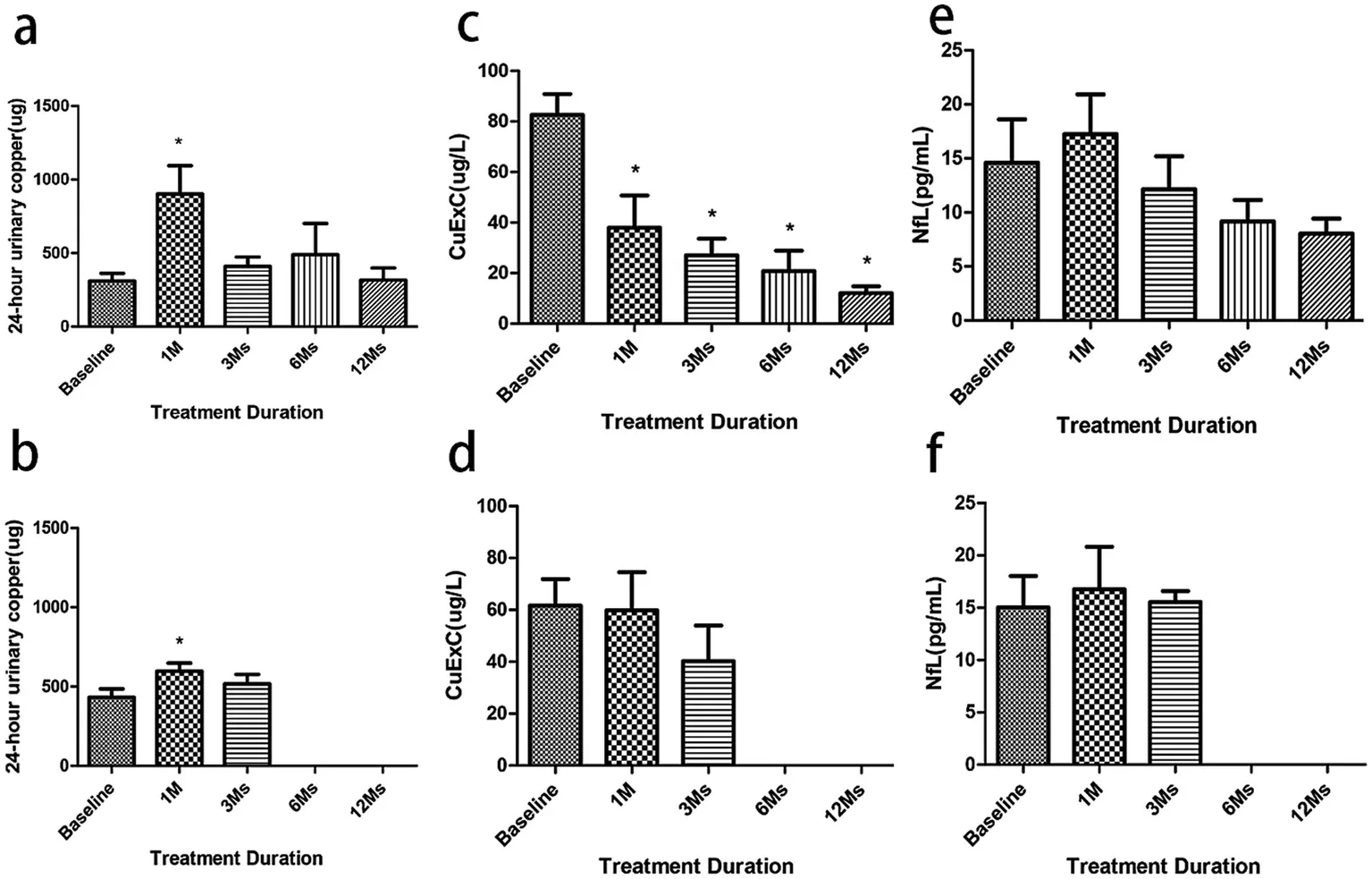
Longitudinal changes in copper metabolism parameters and plasma NfL during treatment. **(a)** In the stable group, 24-h urinary copper excretion increased at 1 month and then gradually declined through 12 months. **(b)** In the worsening group, urinary copper excretion increased significantly at 1 month compared with baseline. **(c)** Serum CuEXC levels in the stable group declined steadily throughout treatment. **(d)** In the worsening group, CuEXC levels did not decrease significantly at 1 month, with only a slight reduction at 3 months. **(e)** In the stable group, plasma NfL levels increased mildly at 1 month, then declined at 3, 6, and 12 months. **(f)** In the worsening group, plasma NfL levels increased slightly at 1 and 3 months compared with baseline. *p < 0.05.

**Figure 2:**
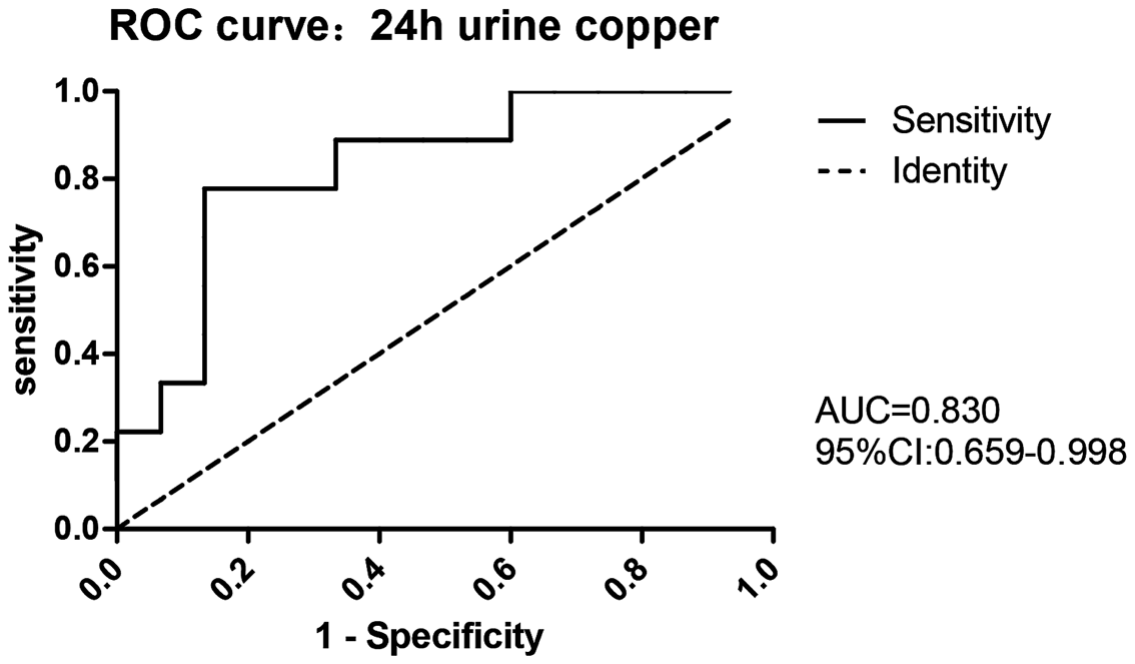
Twenty-four-h urinary copper had good diagnostic performance for disease worsening, with an AUC of 0.830. The optimal cutoff value was 473.85 μg/day, with a sensitivity of 77.8% and a specificity of 86.7%.

### Serum exchangeable copper (CuEXC)

CuEXC in stable patients showed a sustained, progressive decrease from baseline 82.6 (53.6–117.7) to 37.9 (6.2–87.5) at 1 month (*p* = 0.017), 27.0 (6.7–50.1) at 3 months (*p* < 0.001), and 12.0 (5.9–22.1) μg/L at 12 months (*p* < 0.001) (Figure 1**c**). In contrast, worsened patients had no significant reduction at 1 month (59.8 [39.9–88.5] vs. baseline 61.6 [35.9–123.2], *p* = 0.925), and only a modest, non-significant decline at 3 months (40.2 [17.1–64.8], *p* = 0.282) (Figure 1d). No acute CuEXC spikes were observed in either group.

### Plasma NfL

NfL data were available for 18 patients at 1 month. Both groups showed a mild, non-significant rise at 1 month (stable: 14.6 → 17.2 pg./mL, *p* = 0.327; worsened: 15.0 → 16.8 pg./mL, *p* = 0.603) (Figures 1**e**,**f**). Thereafter, NfL in stable patients trended downwards (to 8.0 pg./mL at 12 months, *p* = 0.35 vs. baseline), while in worsened patients it remained slightly elevated at 3 months (15.6 pg./mL). Between-group differences were not statistically significant at any timepoint (all *p* > 0.2).

### Follow-up after neurological worsening

Five worsened patients received intravenous DMPS and reported improvement by telephone; two switched to oral DMSA with no change; two were lost to follow-up.

## Discussion

In this retrospective cohort of treatment-naïve patients with neurological WD who received an ultra-low starting dose of penicillamine (125 mg/d) with slow escalation, early neurological worsening occurred in 36% of patients, all within the first 3 months. Despite the conservative regimen, this rate remains within the 10–50% range reported in historical cohorts on higher doses (1–3), suggesting that even substantial dose attenuation does not eliminate the risk. The key finding is that a 1-month 24 h-urinary copper >473.85 μg/d, identified by ROC analysis, showed favorable discrimination for subsequent clinical stability (sensitivity 77.8%, specificity 86.7%). This threshold represents an exploratory biomarker hypothesis for identifying patients with adequate response to low-dose chelation and requires prospective validation. Although failure to reach this level despite treatment may suggest insufficient copper removal and a higher risk of neurological worsening, prospective multicenter cohort studies are warranted to validate these observations prior to any clinical application.

The CuEXC trajectory provides mechanistic insight. Stable patients showed a rapid, sustained decline in CuEXC from month 1 onward, reflecting effective chelation of the toxic copper pool. In contrast, worsened patients showed no significant CuEXC reduction at 1 month and only a modest, non-significant decrease at 3 months, paralleling their blunted urinary copper rise. The worsening group exhibited no significant NfL elevation at the corresponding time points. We cannot distinguish whether copper depletion preceded neurological worsening, or whether neurological deterioration induced secondary changes in copper metabolism or therapeutic adjustments that subsequently altered CuEXC. Nevertheless, our findings do not support classical copper redistribution as the dominant mechanism underlying early neurological worsening in this cohort—the hypothesis posits that penicillamine mobilizes hepatic copper into the circulation and exacerbates neuronal injury (5–7). If redistribution were the dominant mechanism, we would expect a transient CuEXC spike preceding worsening, but our patients lacked such spikes. Instead, when considering the full longitudinal urinary copper and CuEXC trajectory, our data support the hypothesis that early deterioration is more closely linked to inadequate chelation efficacy in a subset of patients, potentially leading to persistent toxic copper burden and ongoing neurodegeneration.

Plasma NfL is a sensitive biomarker of neuroaxonal damage (8). In this study, both the stable and worsened groups showed mild, transient NfL elevation at month 1 after treatment initiation, reflecting acute neuronal stress induced by early penicillamine chelation. Importantly, no significant intergroup differences in NfL levels were identified at any time point. Additionally, no statistically significant within-group changes in NfL were observed in either cohort throughout follow-up. Although a subtle numerical decrease in NfL was present in stable patients alongside improved UWDRS scores, this mild trend did not reach statistical significance and should not be overinterpreted. The lack of robust NfL changes in the worsening group may be attributable to the relatively short follow-up and limited sample size of this retrospective cohort (9, 10). Overall, the present data indicate that NfL has limited utility as an early predictive biomarker for neurological outcomes in patients receiving ultra-low-dose penicillamine therapy.

Our findings have direct clinical implications. The persistence of neurological worsening despite ultra-low-dose initiation suggests that dose reduction alone cannot fully prevent this complication. Instead, early identification of patients with insufficient copper removal may complement individualized management. The 1-month urinary copper threshold may serve as a preliminary, widely accessible tool to inform early treatment considerations: patients below 473.85 μg/day might warrant evaluation for alternative chelators, whereas those above could be monitored with continued use of the current regimen. This approach could individualize therapy and reduce the anxiety associated with “watchful waiting.” However, the optimal titration strategy for non-responders remains undefined.

Several limitations should be acknowledged. The single-center, retrospective design and small sample size limit statistical power and generalizability. The lack of a control group receiving conventional dosing precludes direct comparison of safety and efficacy. The concomitant use of zinc may confound results and should be addressed in future comparative studies. Treatment switches truncated follow-up in the worsened group, preventing long-term biomarker assessment. Additionally, CuEXC and NfL data were incomplete at some timepoints due to the retrospective nature.

In conclusion, even ultra-low dose penicillamine does not eliminate early neurological worsening, but a 1-month urinary copper threshold can identify patients at lower risk. Inadequate chelation, rather than copper redistribution, appears to underlie deterioration in most cases. Larger prospective studies are needed to validate this threshold and to refine biomarker-guided algorithms for early intervention.

## Data Availability

The datasets presented in this study can be found in online repositories. The names of the repository/repositories and accession number(s) can be found in the article/supplementary material.
